# Illustrations of Vicarious Diseased Action

**Published:** 1855-05

**Authors:** Freeman Knowles

**Affiliations:** Prof. of Theory and Practice of Medicine in the Iowa Medical Department


### ILLUSTRATIONS OF VICARIOUS DISEASED ACTION.

BY FREEMAN KNOWLES, M. D.,

Prof. of Theory and Practice of Medicine in the Iowa Medical Department.

It is among the important duties which every practitioner of medicine owes to the profession, to contribute all important facts and suggestions that may fall under his own observation. It is only by such a course that the profession can make that progress in the procuration of facts and the developmeunt of principles, which is to guide us in our daily conflicts with the protean forms of disease that afflict our race. When we reflect upon the constant mutations that the diseases of our own State and locality are undergoing, and the complicated character too often met with, in what were formerly our most simple forms of disease, it becomes perfectly apparent that the only course which duty and professional success point out, lies in investigation and scrutiny; and that too with unremitting diligence and fidelity, each reporting for the good of all, every important fact connected with new forms of disease, as well as any interesting departure from those of common occurrence.

There are no classes of disease more perplexing to the young practitioner than those which depend upon some remote cause, acting upon vicarious principles. In such cases the function of one organ becomes deranged, in its efforts to perform that of another; and this state of things may continue for months, baffling every form of treatment adopted, so long as the treatment is directed to the consequence of disease, and not to the cause itself. To illustrate the foregoing position, we give a few cases:

CASE I.—Mrs. E——, Nervo-sanguine temperament, had been confined with her first child some four months previous to my being called to see her; found upon enquiry that she was troubled with chronic diarrhœa; tongue flabby, with small yellow blisters about the lips and edges; a brown fur along the centre; evacuations liquid; ten or twelve in the twenty-four hours; color of dejections, dirty white, exhibiting the appearance of yeast in a state of fermentation; had been under the care of two practitioners for about two months. She had taken various prescriptions for nursing sore mouth, as chlorate of potassa, nitrate of silver, sulphate of iron, &c. I was led from the appearance of skin and color of dejections, to believe that the source of the difficulty existed in the liver. Accordingly, upon examination, I found the right hypochondrium enlarged and extremely tender to the touch; pulse small, 85 beats per minute; respiration 19. Prescribed as follows:

| | | | |
|---|---|---|---|
| ℞ | Hydrarg. Protochlor. | fifteen grains. | |
| | Cretæ Preparat. | twenty-five grains. | |
| | Pulv. Opii. | five grains. | M. |

Make into five powders. One powder every three hours; blister 4 by 7 inches over region of liver. Saw patient next morning; had had three exacuations from the bowels, dark and moderately consistent; blister had done well; mouth much better. Prescribed turpentine emulsion every three hours, with pill every night and morning, composed as follows:

℞ Ext. Taraxacum.
Ext. Gentian.
Rad. Sanguinaria, each equal parts.
Opium, half grain, to each pill.

Treatment continued 4 days, at which time, tongue was well; pulse 72; respiration 18; three healthy alvine evacuations in 24 hours. At this time, ordered the emulsion three times per day, with a pill only at night; patient rapidly recovered.

CASE II.—Was called to see Mrs. D——, aged about 30, sanguine bilious temperament; had been laboring under chronic diarrhœa for eleven months; had been under the care of several physicians, taken various prescriptions; had been twice ptyalized without benefit; much emaciated; tongue pale and flabby; pulse feeble; skin cool. Upon enquiring, found that she had labored under deficient catamenial secretion since sometime before the occurrence of her diarrhœa; appetite variable. In this case, the functions of the liver and stomach seemed to be correctly, though feebly performed, while the increased peristaltic action of the bowels carried off their contents too rapidly for the due performance of the lacteal function, and hence the emaciation. Prescribed in this case

℞ Sulphate of Iron
Gum Myrrh
Rad. Sanguinaria, each, half a drachm.
Powdered Opium, fifteen grains.

Mix, and make into thirty pills. One pill to be given every morning, noon, and night, for ten days, after which, patient had a full healthy catamenial secretion, from which time, she rapidly recovered.

In the above case, we find the patient relieved, as soon as the catamenial function is restored, although the affection had resisted every species of treatment directed to the diseased condition of the bowels. So of the former case, when the function of the liver was restored, the apthous condition of the mouth, as well as the abnormal alvine evacuations, subsided, proving conclusively, that the diarrhœa was a consequence or symptom of another deranged function.

CASE III.—Was called to Mrs. G——. She had been confined some two months previously; convalesced rapidly after confinement. About six weeks after her delivery, she was attacked with diarrhœa, ushered in with a chill, after which no rigors appeared. Two weeks after the attack, I was called to prescribe for her; found the patient with skin cool; pulse 90; tongue covered with brown fur; no tenderness over the abdomen, or the right or left hypochondrium; evacuations dirty brown; urine natural, as to quantity and quality; secretion from the breasts nearly suspended. Prescribed as follows:

| | | |
|---|---|---|
| ℞ | Hydrar. Cum Creta, | one drachm. |
| | Pulv. Doveri | half a drachm. |
| | " Camphor | " " |
| | Cretæ prepar. | one " |

Mix, and make into ten powders, one to be taken every three hours, until the evacuations were suspended. Called next evening; little or no change. Prescribed,

| | | |
|---|---|---|
| Acetat. Plumbi. | half drachm. | |
| Pulv. Opii. | ten grains. | |
| Creta prep. | twenty-five grains. | M. |

Make six powders, one to be given every four hours. Saw patient next day; little or no amendment. Prescribed emulsions of the terebinthinates, astringents, mercury to ptyalism, all to no permanent purpose, for nearly two months. One day, I discovered that the patient had slight yawning every morning and evening, although followed by no reaction. Prescribed Sulph. Quinine, half drachm; Dover, twenty grains; divided into six powders; give one every two hours; next morning the patient had a severe paroxysm of ague, and from that time, every vestige of the disease disappeared and the patient rapidly recovered.

This case fully illustrates some of the peculiarities of our western diseases. Here was a case of diarrhœa resisting all the remedies usually given in that disease and yielding readily to the quinine, proving, in the first place, the miasmatic origin of the disease, and secondly, how very obscure are some miasmatic affections of our climate.

How carefully, then, ought the practitioner in paludal districts, to watch this subtle element which enters so largely into all the different forms of disease in such localities. It matters not what is its form, whether pneumonia, peritonitis, typhoid fever, or any other inflammatory affection, this influence is always present, complicating both the symptomatic phenomena, as well as the treatment. Hence the importance of studying carefully the complication, and the diagnostic signs that warrant the use of quinine in inflammatory affections.

